# Role of Transport Proteins for the Renal Handling of L-Arginine and Related Derivatives

**DOI:** 10.3390/ijms26167899

**Published:** 2025-08-15

**Authors:** Lorenz A. Scherpinski, Jörg König, Renke Maas

**Affiliations:** 1 Institute of Experimental and Clinical Pharmacology and Toxicology, Friedrich-Alexander-Universität Erlangen-Nürnberg, 91054 Erlangen, Germany; lorenz.scherpinski@fau.de (L.A.S.); joerg.koenig@fau.de (J.K.); 2 FAU NeW Research Center New Bioactive Compounds, Friedrich-Alexander-Universität Erlangen-Nürnberg, 91054 Erlangen, Germany

**Keywords:** transport, kidney, proximal tubule, L-arginine, L-homoarginine, ADMA, SDMA

## Abstract

L-arginine and its derivatives L-homoarginine, asymmetric dimethylarginine (ADMA), and symmetric dimethylarginine (SDMA) show distinct (patho-) physiological properties as well as a differential renal handling. L-arginine and L-homoarginine have a lower renal clearance and are largely retained (i.e., reabsorbed) as compared to ADMA and SDMA, which are relatively enriched in the urine and excreted. To obtain a more complete picture of what is known regarding transport proteins involved in renal reabsorption and secretion of these substances, a comprehensive literature review and search of cell-specific gene expression databases were performed. Five transport proteins known to transport L-arginine and its derivatives were included, and the data available regarding their tubular expression pattern and their transport characteristics, as well as experimental and clinical data regarding their possible impact on the renal handling of L-arginine and its derivatives, are presented and discussed in a structured narrative review. Based on their transport properties and links to clinical phenotypes, b^0,+^AT-rBAT and y^+^LAT1-4F2hc were identified as the most promising candidates to explain a significant part of the observed differential renal handling. This also makes them promising candidates for further investigations as mediators of possible adverse and beneficial drug effects involving L-arginine, L-homoarginine, ADMA, and SDMA.

## 1. Introduction

L-arginine and its derivatives L-homoarginine, asymmetric dimethylarginine (ADMA), and symmetric dimethylarginine (SDMA) play an important role in cardiovascular health [1,2,3,4,5,6,7], with the L-arginine-nitric oxide (NO) pathway as a major connecting mechanism regulating endothelial function and vascular tone [8]. Elevated ADMA or SDMA plasma concentrations predict adverse cardiovascular outcomes and death [5,9,10]. A proposed contributing mechanism is their inhibitory effect on NO synthesis, either by competitively inhibiting nitric oxide synthase (NOS) [5,10] or by interference with protein-mediated arginine transport into cells [11,12]. These mechanisms could be the reason why increased plasma concentrations of ADMA and SDMA correlate with cardiovascular disease and mortality [3]. In contrast, L-homoarginine has been associated with protective cardiovascular effects, and reduced plasma concentrations have been linked to adverse outcomes in both kidney and cardiovascular diseases [7,13,14,15].

The risk of cardiovascular disease is increased by two to four times in patients with chronic kidney disease (CKD) [16], and accumulation of uremic toxins, including ADMA and SDMA, or loss of protective factors like L-homoarginine, have been implicated as contributing factors [2,3,5]. The kidney is a main organ of homeostasis for L-arginine, L-homoarginine, ADMA, and SDMA. However, despite their similar chemical structures, these four molecules are processed differently by the kidneys, which is reflected by their renal plasma clearance values: while L-arginine and L-homoarginine exhibit low clearance rates (0.12–0.27 mL/min [17,18,19] and 1.06–1.50 mL/min [17,18], respectively), indicating substantial reabsorption or retention, ADMA and SDMA show a higher clearance (77.50–85.74 mL/min [17,20] and 80.10–81.73 mL/min [17,20], respectively), pointing to predominant urinary excretion. These differences in clearance suggest distinct renal handling mechanisms that extend beyond glomerular filtration alone.

Renal plasma processing involves not only filtration, but also tubular transport, local synthesis, and metabolic transformation. L-arginine and L-homoarginine are not only reabsorbed after glomerular filtration into proximal tubule cells but are also synthesized intracellularly and released into blood [21,22]. In contrast, the methylarginines ADMA and SDMA, which primarily remain in the urine after glomerular filtration, are also metabolized by enzymes in proximal tubule cells, requiring uptake from either the tubular lumen or the peritubular capillaries [6,23,24,25,26]. The uptake and release of L-arginine and its derivatives into and out of renal tubular cells are mediated by transport proteins [12,27,28,29,30,31].

So far, a total of 19 different transport proteins have been shown or proposed to mediate the transport of L-arginine and its derivatives (see review by Banjarnahor et. al. [30]). No transport protein has yet been identified that facilitates the transport of L-homoarginine, ADMA, or SDMA without also transporting L-arginine. However, only some of these 19 transporters are expressed in the kidney and are, therefore, likely to be involved in renal handling. Previous suggested models describing the transporter environment in the proximal tubule focused only on L-arginine and did not cover the mechanisms behind the distinct renal handling of the arginine derivatives such as ADMA, SDMA, and L-homoarginine [32,33].

Given their distinct role as nutrients, signaling precursors, and protective factors in the case of L-arginine and L-homoarginine, as opposed to possible detrimental effects attributed to ADMA and SDMA as uremic toxins, a differential renal handling of these chemically related substances comes as no surprise. However, to our best knowledge, the underlying transport mechanisms have never been systematically assessed until now.

The scope of this review is the handling of L-arginine and its derivatives by transport proteins, expressed in the human kidney, with a focus on transporters expressed in proximal tubule cells. Recent findings and advances in single-cell transcriptomics allow us to reassess the role of these transporters in the physiology and renal handling of L-arginine and its cardioactive derivatives in new detail.

## 2. Cardioactive Arginine Derivatives

A general overview of L-arginine and its derivatives is compiled in Table 1.

### 2.1. L-Arginine

As a semi-essential amino acid, L-arginine is part of protein synthesis and multiple metabolic and signaling pathways [21,34]. For its daily supply, the body relies on diet (4–5 g per day [21]), endogenous synthesis (2 g per day [21]), and release from protein turnover. Endogenous L-arginine synthesis primarily occurs in the kidneys from citrulline via argininosuccinate synthase and lyase [21,35,36]. The progression of CKD leads to only minor changes in plasma L-arginine concentrations [4,37,38,39], likely due to compensatory mechanisms such as increased synthesis from citrulline [37,40].

L-arginine is the precursor of the vasodilator NO [8], which is produced by NOS enzymes [41]. NOS metabolize L-arginine or L-homoarginine to NO and L-citrulline or L-homocitrulline, respectively [8,42]. Consequently, L-arginine supplementation and a high-protein diet were investigated as possible therapies for high blood pressure and showed significant results in short-term interventions [43,44,45,46]. Long-term supplementation of 9 g L-arginine daily was associated with increased mortality in a single study [47]. However, long-term supplementation of L-arginine may have only a limited impact on plasma L-arginine, possibly due to counterregulatory mechanisms. Supplementation of its metabolic precursor citrulline may be much more efficient with respect to elevating plasma L-arginine [48].

### 2.2. L-Homoarginine

L-homoarginine is a non-proteinogenic cationic amino acid. It is a dietary component but also synthesized from lysine by the enzyme arginine:glycine amidinotransferase (AGAT) [49], which is expressed in multiple organs but to the greatest extent in the kidney [49]. It has been characterized as an independent protective marker for mortality in coronary heart disease and hemodialysis patients [7,15]. The cardioprotective properties have, in part, been attributed to its role as a secondary substrate of NOS for NO synthesis [42]. Similar to L-arginine, it is part of the glomerular filtrate and largely reabsorbed in the renal tubules [17].

With advancing renal impairment, homoarginine plasma concentrations decrease [2,13]. However, whether the inverse correlation of its plasma concentration and the estimated glomerular filtration rate (eGFR) of the kidney depends on a decline in renal reabsorption or synthesis is not fully understood.

### 2.3. ADMA

ADMA serves as a direct inhibitor of NOS [5,10]. Elevated plasma concentrations of ADMA are independently associated with elevated total and cardiovascular mortality [3], of which inhibition of NOS has been proposed as a possible mechanism [3,5]. ADMA originates from protein degradation of previously methylated arginine [50,51]. Around 80% of ADMA is metabolized by the enzymes dimethylaminohydrolase 1 (DDAH1) [9,52] and, to a minor degree, by alanine-glyoxylate aminotransferase 2 (AGXT2) [53]. DDAH1 is expressed widely in different tissues, including the kidney, pancreas, and liver [52]. AGXT2 is mainly expressed in the kidney and the liver [54]. The remaining ADMA is renally eliminated [25,55]. ADMA was originally characterized as a uremic toxin in hemodialysis patients and patients with kidney failure [5]. To investigate the impact of the eGFR on the renal plasma clearance of ADMA in humans, Ronden et al. took renal artery and vein samples of hypertension patients with mild to moderate renal insufficiency [25]. They could show that the eGFR is not independently associated with the renal plasma clearance of ADMA, with only a minor decrease in the renal plasma clearance between the different eGFR groups [25]. The persistence in renal plasma clearance independent of the eGFR indicates that ADMA is taken up from the blood into proximal tubule cells and metabolized there instead of being filtered. Additionally, Carello et al. reported that ADMA plasma clearance persisted after total nephrectomy in rats while SDMA levels were rising, indicating that renal ADMA elimination can be compensated by hepatic DDAH1 [56]. It was also shown that cirrhosis patients have elevated ADMA concentrations that are lowered again after compensated cirrhosis was accomplished [57].

**Table 1:** Key characteristics of L-arginine and its derivatives in healthy and CKD patients (adapted and extended from Banjarnahor et al. [30]).

	L-Arginine	L-Homoarginine	ADMA	SDMA
Structure	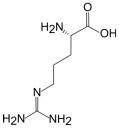	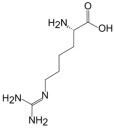	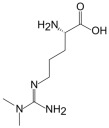	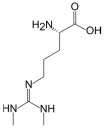
Source or synthesis	Endogenous: via biosynthesis between 9.2 and 16 µmol × kg^−1^ × h^−1^ equals ~2.8–5 g/day in male adults [58,59]	Endogenous: synthesis by the enzyme AGAT [49]	Endogenous: hydrolysis of proteins after asymmetric methylation (~60 mg/day) [23,50,51]	Endogenous: hydrolysis of proteins after asymmetric methylation [23,50,51]
	Diet: approx. 5 g/day [60]	Diet: unknown proportion	Diet: unknown proportion	Diet: unknown proportion
Metabolism and elimination	Major enzymes: AGAT, NOS (3 isozymes), arginases (2 isozymes), and L-arginine decarboxylase [21,34]	Major enzymes: AGXT2 [61]; arginases and NO-Synthases [42,62]	Major enzymes: DDAH1 accounts for >80% of the metabolic elimination [9,52]; AGXT2 [53] Elimination: renal excretion ~20% [25,55]	Major enzyme: AGXT2 (mildly elevated plasma concentration in genetic AGXT2 deficiency) [53] Elimination: primarily by renal excretion [25,56]
Protein binding	<4% [63]	No data found	8% [23]	9% [23]
Plasma concentration mean values [µmol/L]	83–153 [4,20,64,65,66,67,68]	1.19–2.5 [65,69,70]	0.23–0.67 [4,26,65,66,67,71,72]	0.15–0.53 [4,26,65,66,72,73]
Renal clearance [mL/min]	0.12–0.27 [17,18,19]	1.06–1.50 [17,18]	77.50–85.74 [17,20]	80.10–81.73 [17,20]
Effect of impaired renal clearance on plasma concentration	Unchanged [4,37,38,39]	Lowered ~30% [13]	Elevated ~10–100% [4,5,26,66,74]	Highly elevated ~50–1000% [4,26,66,74]

ADMA: asymmetric dimethylarginine; SDMA: symmetric dimethylarginine; AGAT: L-arginine:glycine amidino transferase; NOS: NO synthases; AGXT2: alanine —glyoxylate aminotransferase 2; DDAH1: Dimethylarginine dimethylaminohydrolase 1.

### 2.4. SDMA

For a long time, SDMA was considered biologically inert [5]. However, recent studies could show that SDMA can increase monocytic reactive oxygen species production [75], activate NF-κB [76], influence several immunologically relevant transcription factors [77], and is a weak inhibitor of L-arginine transport [12,29,78]. Like ADMA, SDMA is produced during protein degradation [23,50,51]. SDMA is poorly metabolized except in small amounts by AGXT2 [53], and single-nucleotide polymorphisms in the *AGXT2* gene in mouse and humans are associated with a mild increase in plasma concentration [53,79]. However, the main route of elimination is via the kidneys [25,56], where it correlates stronger with eGFR than ADMA [4]. Therefore, multiple studies investigated SDMA as a biomarker for CKD, where it showed promising results in predicting CKD progression [4,5,36,74,80]. In a chronic SDMA infusion mouse model, Veldink et al. demonstrated that a significant increase in SDMA plasma concentration from 0.26 ± 0.1 to 3.49 ± 1.66 µM did not result in changes in renal or cardiac function [81]. These findings suggest that the close inverse correlation between the plasma concentrations of SDMA and the eGFR may primarily drive its association with all-cause mortality, aside from its weak NOS-inhibiting effects [42].

## 3. Renally-Expressed Transport Proteins Related to L-Arginine Transport

**Figure 1:**
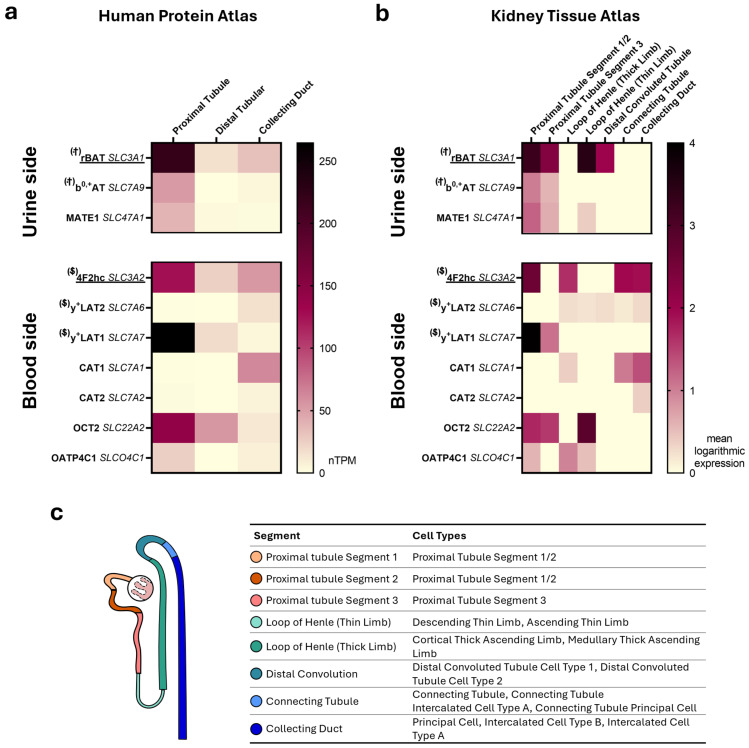
Gene expression of transport proteins mediating L-arginine transport in the renal tubule determined by single-cell RNA data. Proteins are grouped based on their location, facing the urine or blood. Proteins marked with an † or an $ form a heterodimer consisting of an anchor protein and the functional transporter; the underlined protein functions as anchor protein. (**a**) Data from the Human Protein Atlas (HPA)—the database contains genome-wide single-cell RNA data for 76 cell types. Regarding the tubular system of the kidney, three different cell types are supported: proximal tubule, distal tubule, and collecting duct. Expression data are given in transcripts per million [92,94,95,96,97,98,99,100,101,102]. (**b**) Kidney Tissue Atlas (KTA) provides data from three cohorts: healthy, chronic kidney disease, and acute kidney injury. The data were clustered not only by cell type markers but also by cell status markers (e.g., enriched cell cycling genes), offering a high single-cell resolution of the tubular system, and containing 14 cell types. For an improved overview, different cell types were combined into seven segments. The highest expression of one cell type per segment is shown. Only data from the healthy cohort without enriched cell status markers were used for this expression analysis. Data are provided as mean logarithmic expression https://www.kpmp.org (accessed on 4 March 2025) [93,103]. (**c**) Schematic representation of a nephron and its segments and cell type classification by the KTA.

A total of 19 transport proteins have been identified mediating the transport of L-arginine [30,78]. However, only eight transport proteins are expressed in kidney cells: CAT1 (gene: *SLC7A1*), CAT2 (gene: *SLC7A2*), y^+^LAT1-4F2hc (gene: *SLC7A7/SLC3A2*), y^+^LAT2-4F2hc (gene: *SLC7A6/SLC3A2*)*,* b^0,+^AT-rBAT (gene: *SLC7A9/SLC3A1*), OCT2 (gene: *SLC22A2*), OATP4C1 (gene: *SLCO4C1*), and MATE1 (gene: *SLC47A1*), making them the only ones likely to be relevant to renal handling [82,83,84,85,86,87,88,89,90,91]. Most of the expression data available, so far, were based on whole organ analysis instead of single-cell assays, causing uncertainty about whether these proteins are also expressed in the renal tubular system. However, in recent years, several comprehensive database projects aimed to close this gap by providing publicly available single-cell RNA (scRNA) data sets. For the present analysis the scRNA expression data provided by the Human Protein Atlas (HPA) [92,93,94,95,96,97,98,99,100,101,102] and the Kidney Tissue Atlas (KTA) (The KTA data were generated by the Kidney Precision Medicine Project. https://www.kpmp.org (accessed on 4 March 2025) [93,103]) were used to assess and visualize which transport protein is expressed in which part of the tubular system (Figure 1).

The expression patterns were comparable in both databases. Transport proteins located in the apical membrane were predominantly expressed in proximal tubule cells, enabling only these cells to reabsorb L-arginine or its derivatives. Other segments of the tubular system showed only basolateraly expressed transporters, which can take up L-arginine from the blood, e.g., for protein synthesis. In proximal tubule cells, scRNAs of the following apically localized transport proteins were detected: b^0,+^AT-rBAT, and MATE1, and at the basolateral side y^+^LAT1-4F2hc, OCT2, and OATP4C1. The KTA has a higher resolution analysis of the tubular system compared to HPA and separates the proximal tubule further into multiple segments. These data indicate that *SLC7A9/SLC3A1* and *SLC7A7/SLC3A2* are mainly expressed in the first two proximal tubule segments.

For CAT1, CAT2, and y^+^LAT2, we found no evidence of expression in the proximal tubule. In previous works, CAT1 had been proposed to be part of the renal arginine homeostasis [33,104]. However, both single cell expression databases we utilized show that the *SLC7A1* gene, encoding CAT1, is not expressed in the proximal tubule but rather in the collecting duct. These findings are in line with reports of CAT1-associated NO production in the collecting duct of rats [105,106].

**Figure 2:**
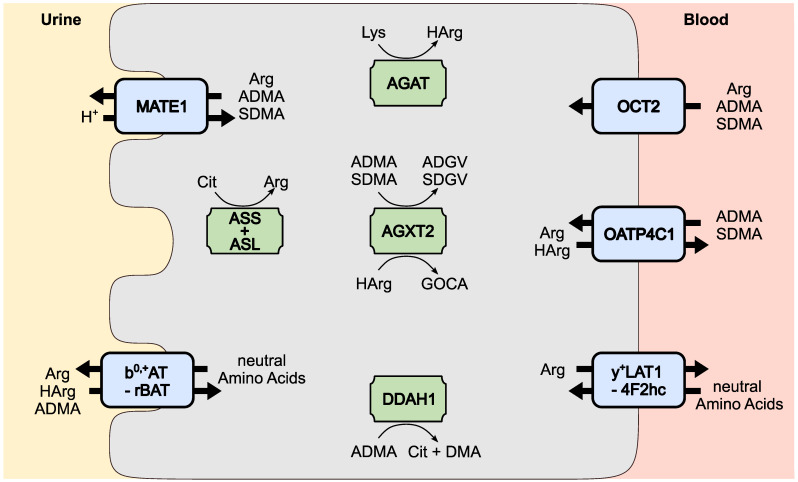
Renal handling of L-arginine and L-arginine derivatives in proximal tubule cells. Transport proteins mediating the uptake and/or export of substances are shown in blue, and enzymes involved in metabolism are shown in green. Abbreviations: arginine (Arg), arginine:glycine amidinotransferase (AGAT), Alanine-Glyoxylate Aminotransferase-2 (AGXT2), α-keto-δ asymmetric dimethyl–guanidino valeric acid (ADGV), argininosuccinate lyase (ASL), argininosuccinate synthase (ASS), asymmetric dimethylarginine (ADMA), citrulline (Cit), dimethylaminohydrolase 1 (DDAH1), dimethylamine (DMA), 6-guanidino-2-oxocaproic acid (GOCA), homoarginine (HArg), lysine (Lys), α-keto-∆ symmetric dimethyl–guanidino valeric acid (SDGV), and symmetric dimethylarginine (SDMA).

The expression pattern narrows the number of potential transport proteins responsible for arginine derivative homeostasis down to five candidates, which are further described in more detail (Figure 2).

### 3.1. y^+^LAT1 (SLC7A7) and 4F2hc (SLC3A2)

The y^+^L amino acid transporter 1 (y^+^LAT1) is encoded by the *SLC7A7* gene. It is one of the two members of the y^+^L transport system. Together with the anchor protein 4F2hc (gene *SLC3A2*), it forms a heterodimeric protein, in which the 4F2hc is necessary for plasma membrane localization [88,107]. Both proteins are regulated by mTOR, which is activated, among other things, by intracellular amino acid deficiency [108]. The transporter is located in the basolateral membrane of proximal tubule cells, especially in segment 1/2 (Figure 1) and in intestinal cells [82,88], with protein expression confirmed by immunohistochemistry in rodent kidney tissue [104]. In these cells, y^+^LAT1-4F2hc acts as an exchange protein that mediates a cationic amino acid efflux coupled to a Na^+^ and neutral amino acid influx [32,109,110]. This results in an approximately five-fold gradient between cationic (lower in the cytosol) and neutral (higher in the cytosol) amino acids [32,110]. In the absence of Na^+^, y^+^LAT1-4F2hc loses its affinity to neutral amino acids and transports only cationic amino acids, which are transported independently of Na^+^ [82]. Transport kinetic studies in monocyte-derived macrophages showed an apparent K_m_ value of 182 µM for L-arginine (Table 2) [111]. For the arginine derivatives, no kinetic parameters have been identified so far.

Nevertheless, two case reports give insights into possible interactions between L-homoarginine, ADMA, and y^+^LAT1-4F2hc. In a patient with lysinuric protein intolerance (LPI), a disease caused by mutations in the *SLC7A7* gene, Kato et al. observed elevated L-homoarginine urine concentrations by a factor of 63 to 563 as compared to controls [69]. This indicates that the release of L-homoarginine into blood after renal reabsorption is largely mediated by y^+^LAT1-4F2hc [69]. LPI patients also have lower L-arginine plasma concentrations and elevated urinary L-arginine concentrations [69]. This may explain why LPI is also associated with reduced NO production and endothelial dysfunction [116,117]. A connection between y^+^LAT1-4F2hc and ADMA was reported by Closs et al. [112]. They observed an increased intracellular ADMA concentration in a patient with reduced y^+^LAT1 expression [112]. They hypothesized that y^+^LAT1 expression mediates ADMA efflux out of the cells and that this mechanism is an additional factor contributing to reduced endothelial function in LPI, since ADMA is a known inhibitor of eNOS [5,112]. For further reading on the role of y^+^LAT1 in other tissues, we recommend the following reviews [82,110].

### 3.2. b^0,+^AT (SLC7A9) and rBAT (SLC3A1)

b(^0,+^)-type amino acid transporter 1 (b^0,+^AT) is the active part of the heterodimeric protein b^0,+^AT-rBAT, which is encoded by the genes *SLC7A9* and *SLC3A1.* rBAT alone has no transport activity; it functions as an anchor protein for directing b^0,+^AT to the apical membrane and is joined to b^0,+^AT by a disulfide bridge [82,118]. Similar to y^+^LAT1-4F2hc, the gene expression of b^0,+^AT-rBAT is regulated by mTORC1 [108]. Both genes are expressed in the proximal tubule, although *SLC3A1* expression is not limited to this segment (Figure 1). This expression pattern is caused by a second light chain AGT-1 encoded by the *SLC7A13* gene, which also forms a heterodimer with rBAT mainly in the S3 segment and distal convoluted tubule [119,120]. However, western blot data from rodent studies confirmed b^0,+^AT-rBATs limitation to the proximal tubule [121]. b^0,+^AT-rBAT acts as an obligatory heterodimeric amino acid exchanger [82]. Under physiological conditions, b^0,+^AT-rBAT mediates the uptake of cationic amino acids or cysteine in exchange for neutral amino acids, independent of sodium [122]. The physiological transport direction is probably based on an intracellular negative membrane potential [82], but, hypothetically, it can also mediate the transport in the opposite direction if the conditions are altered. Why the transporter mediates cationic amino acid and cysteine uptake is not fully understood since cysteine tends to have an anionic character at neutral pH. However, the cryo-EM structure of b^0,+^AT revealed that the transporter has, next to the classical binding pocket 1, an additional binding pocket 2 [123], while other heterodimeric transporters, such as LAT1 (*SLC7A5*), have only one binding pocket [124,125]. It is assumed that the second pocket enables this broader substrate spectrum. Different kinetic parameters (K_m_ values) for L-arginine have been reported, depending on the cell model used for analysis, ranging between 108 µM and 512 µM (Table 2) [27,113,126]. Banjarnahor et al. determined a K_m_ value of 197 µM for L-homoarginine in Madin–Darby canine kidney cells (MDCK) overexpressing human b^0,+^AT-rBAT [27]. The same study also investigated ADMA as a substrate of b^0,+^AT-rBAT [27]. It was shown that ADMA is also transported; however, due to a lack of saturation, a K_m_ value could not be determined [27]. As mentioned before, b^0,+^AT-rBAT is theoretically capable of mediating L-arginine transport in both directions. Using chicken jejunal cells, K_m_ values of ~8 µM for uptake and ~180 µM for efflux were reported for the avian b^0,+^AT-rBAT protein, which highlights the physiologically preferred direction of mediating L-arginine uptake [127,128].

Mutations in b^0,+^AT-rBAT are associated with cystinuria, a disease in which excessive amounts of cysteine, lysine, arginine, and ornithine remain in the urine [129]. Cystinuria is classified as type A (mutations in rBAT), type B (mutations in b^0,+^AT), or type AB (mutations in both) [82,130]. Although several key mutations have been identified, data on genotype–phenotype correlations remain limited [129]. There is a case report describing a specific mutation in the b^0,+^AT binding pocket, which converts the transporter into a specific cationic amino acid transporter [82,131]. Based on the mutation, the degree of cystinuria can differ between patients. Cox et al. reported that urinary excretion of L-arginine changed from 7–35 µmol/24 h in healthy individuals to 1455–8670 µmol/24 h in cystinuria patients [18]. The same study reported a urinary excretion of 15–51 µmol/24 h for L-homoarginine in cystinuria patients, as compared to 1–6 µmol/24 h in controls [18], indicating that L-homoarginine reabsorption from the urine is mainly mediated by b^0,+^AT-rBAT. The elevated amounts of L-arginine and L-homoarginine in the urine of cystinuria patients suggest that b^0,+^AT-rBAT is a major, if not the main, renal mediator for the reabsorption from the urine and is, thus, likely to be one key player in the homeostasis of L-arginine and its derivatives [30]. Whether SDMA is a substrate of the b^0,+^AT-rBAT is still unknown and remains to be investigated. For further reading on the role of b^0,+^AT-rBAT in other tissues, we recommend the following reviews [82,110].

### 3.3. OATP4C1 (SLCO4C1)

The organic anion transporting polypeptide 4C1 (OATP4C1) is encoded by the *SLCO4C1* gene which is mainly expressed in proximal tubule cells (Figure 1) [132], where the protein locates at the basolateral membrane [89]. OATP4C1 is a bidirectional Na^+^-independent transporter and has a broad substrate spectrum, ranging from endogenous compounds such as thyroid hormones, cyclic AMP, to uremic toxins (including ADMA) and xenobiotics like digoxin [29,30,89,133]. ADMA is taken up by OATP4C1 from blood into proximal tubule cells (uptake K_m_ value 232.1 µM) [29,114]. For L-arginine, L-homoarginine, and SDMA, the physiological transport direction is not fully investigated. OATP4C1 can mediate the uptake (K_m_ values: 48.1 µM L-arginine, 49.9 µM L-homoarginine, and 70 µM SDMA) and the efflux of all three substances (Table 2) [29,115]. The K_m_ values of ADMA, SDMA, and L-homoarginine, but not of L-arginine, are above the physiological plasma concentrations, which raises questions about the contribution of OATP4C1 to the homeostasis of these substances [29,115]. However, two animal studies in cats and rats demonstrated a significant decline in *SLCO4C1* mRNA expression in CKD cohorts compared to healthy controls, suggesting that transporter downregulation may be involved in the disease-associated changes in solute handling [134,135]. A possible cause for the downregulation could be indoxyl sulfate, another uremic toxin which accumulates during CKD. It was shown that indoxyl sulfate downregulates OATP4C1 gene expression in a concentration-dependent manner [136]. In addition, Toyohara et al. showed in a rat model that statin administration increased renal *SLCO4C1* mRNA expression, accompanied by a significant rise in ADMA elimination [114]. These findings imply that, despite its relatively high K_m_ value, OATP4C1-mediated ADMA transport may play a relevant physiological role.

### 3.4. OCT2 (SLC22A2)

The organic cation transporter 2 (OCT2) is encoded by the *SLC22A2* gene, which is primarily expressed in the kidney [90,91]. Studies in rodents suggest that the gene expression is regulated by sex hormones like testosterone [137]. In the proximal tubule, OCT2 is located at the basolateral membrane [138], where it mediates the uptake of several cationic molecules [90]. Interestingly, rodent proximal tubule cells also express the closely related transport protein Oct1. Due to the overlapping substrate spectrum of Oct1 and Oct2, Oct2-deficient mice^−/−^ show no changes in renal function compared to wild type animals [139]. However, this compensatory mechanism does not exist in humans and should be considered when comparing human and animal data. The broad substrate spectrum of OCT2 includes endogenous molecules, for example, creatinine, N1-methylnicotinamide, and uremic toxins, as well as several drugs such as metformin, varenicline, or antiretrovirals, which make OCT2 an important site of drug interactions and off-target drug effects [140,141]. OCT2 facilitates the first step in renal secretion of cationic drugs by mediating the drug uptake into proximal tubule cells out of the blood, followed by an efflux into tubular filtrate mediated by MATE1 [142]. As a result, various pharmacologically important drug–drug interactions are associated with OCT2 [143], which is the reason Galetin et al. categorized OCT2 as a clinically important transporter of category A [144]. They further recommended that during drug development, OCT2 interactions should be monitored by changes in the N1-methylnicotinamide levels as an endogenous biomarker of OCT2 [144]. Additionally, several pharmacologically relevant polymorphisms in the *SLC22A2* gene have been identified, most of which are associated with altered drug disposition rather than disease phenotypes [30].

Further characteristics of OCT2-mediated transport include its sodium independency, bidirectionality, and dependence on the membrane potential [142,145]. The cationic substrate preference of OCT2 led to investigations about whether L-arginine, ADMA, or SDMA are also substrates [28,115]. OCT2 is capable of mediating the transport of all three substances; however, the high K_m_ values of >10,000 µM for L-arginine, 900 µM for ADMA, and no saturation reached for SDMA indicate a low affinity and suggests that OCT2 may play a minor role at physiological concentrations (Table 2) [28,115]. Nonetheless, the impact of OCT2-mediated SDMA transport should not be dismissed too readily: although 1 µM SDMA was used in this study, even a 100-fold higher concentration of the OCT2 inhibitor cimetidine (100 µM) inhibited uptake by only 75%, indicating that SDMA is not easily displaced. This may point to a more complex interaction with OCT2 than suggested by the lack of saturation [115]. Currently, there are no available data on OCT2-mediated transport of L-homoarginine. Despite these findings, the physiological relevance of OCT2 in handling endogenous compounds such as methylated arginines remains uncertain. Historically, research on OCT2 has focused on its role in drug disposition. More recently, however, biomarker research has drawn attention to its relevance for the transport of endogenous compounds [146,147]. In vitro studies showed the inhibitory potential of different uremic toxins that accumulate during the progression of CKD, leading in vivo to further changes in drug secretion [141,148]. ADMA was among the substances tested by Cheung et al. as potential inhibitors of metformin uptake; however, even at concentrations 100 times higher than physiological plasma levels, it showed only minimal inhibition (approximately 4%) [148]. However, dimethylamine, the product of DDAH1-mediated ADMA degradation, was identified as an inhibitor [148]. In summary, the role of OCT2 in the homeostasis of L-arginine and its derivatives remains unclear and needs further investigation, particularly concerning the transport of the uremic toxins ADMA and SDMA.

### 3.5. MATE1 (SLC47A1)

The multidrug and toxin extrusion protein 1 (MATE1) is encoded by the *SLC47A1* gene and expressed in several tissues, with the highest expression in the liver and kidney [83]. In the tubular system of the kidney, the expression is restricted to the proximal tubule (Figure 1), where MATE1 is located in the luminal membrane (Figure 2) [83,138]. The transport is sodium-independent but coupled to a H^+^ exchange, making the transport direction dependent on the pH gradient [83]. Under physiological pH conditions, MATE1 predominantly mediates the efflux out of cells, but for experimental studies MATE1 can be analyzed as an uptake transporter by changing the pH of the uptake buffer. Such an experimental setup should only be applied to substances whose formal charge remains unchanged under these modified conditions. MATE1 mediates the renal elimination of multiple endogenous and exogenous substrates like creatinine, trimethylamin-N-oxide (TMAO), or metformin after OCT2-mediated uptake into proximal tubule cells [83,146,149]. Due to its role in drug elimination and potential for significant drug–drug interactions, comparable to OCT2, MATE1 has also been designated a clinically important transporter in category A [144]. Irrespective of drug interactions, high fluctuations in the renal elimination of metformin were observed between patients [150]. Following up on these findings, Kajiwara et al. screened 89 Japanese subjects for MATE1 single nucleotide polymorphisms (SNP) and identified 8 SNPs [151]. Three of these SNPs (A310V, D328A, and N474S) significantly reduced the transport ratio, and one SNP (G64D) caused a total loss of function [151]. Due to the wide range of substrates, Strobel et al. investigated L-arginine and ADMA as potential substrates of MATE1. With increasing alkalinization, the highest uptake ratio for L-arginine (100 µM) and ADMA (1 µM) was observed at a pH of 8.2 with 1.31 and 1.24, respectively (Table 2) [28]. Recently, it was shown that MATE1 can also transport SDMA with a K_m_ value of 1973 µM [115].

Regarding CVD, the role of MATE1 in drug excretion may be more significant than its involvement in the handling of L-arginine and its derivatives, as most studies focus on MATE1’s function in drug transport and drug-induced nephrotoxicity [152]. The known endogenous substrate spectrum of MATE1 is relatively limited [83,146,149]. However, ongoing research into biomarkers may reveal novel interactions with endogenous compounds, similar to findings for OCT2. Despite this, the low transport ratios for L-arginine and ADMA [28], along with the very high K_m_ value for SDMA [115], suggest that these substances are relatively poor substrates for MATE1.

## 4. Conclusions

In this combined assessment of the literature and expression databases, five transport proteins most likely involved in the tubular uptake and release of L-arginine and its derivatives were identified (Figure 1 and Table 2). Taken together, the experimental and clinical data detailed above indicate that, of these, the heterodimeric protein b^0,+^AT-rBAT (encoded by *SLC7A9* and *SLC3A1*) and y^+^LAT1 (encoded by *SLC7A7*) with its anchor protein 4F2hc (encoded by *SLC3A2*) are the most plausible candidates involved in the observed differential renal handling of L-arginine, L-homoarginine, ADMA, and SDMA.

### 4.1. The Role of Overlapping Substrate Specificities of Tubular Transport Proteins

L-arginine and its derivatives are substrates of several transport proteins with different expression patterns in the body. So far, L-arginine and its derivatives have been identified as substrates for 19 transport proteins [30,78]. Newly available single-cell RNA sequencing data confirm expression of OCT2, y^+^LAT1-4F2hc, b^0,+^AT-rBAT, MATE1, and OATP4C1 in the proximal tubule (Figure 1), supporting their role in renal handling of these metabolites [92,93,103]. At first glance, transport of these substances appears to be full of redundancy, as the close chemical properties of L-arginine and its derivatives result in overlapping transport systems (Figure 2). However, these apparent redundancies may serve distinct physiological functions, such as enabling bidirectional transport or ensuring the continuous handling of essential compounds like L-arginine.

The distinct renal handling of the L-arginine derivatives suggests that not all substrates benefit equally from these overlapping systems. A substance that is exclusively filtered by the glomerulus in healthy adults (e.g., inulin) exhibits a renal clearance of around 100–120 mL/min, depending on age [153]. In contrast, the renal plasma clearances of L-arginine (0.12–0.27 mL/min [17,18,19]) and L-homoarginine (1.06–1.50 mL/min [17,18]) indicate efficient reabsorption. By comparison, ADMA (77.50–85.74 mL/min [17,20]) and SDMA (80.10–81.73 mL/min [17,20]) show clearance values closer to that of inulin, suggesting minimal reabsorption and predominant renal elimination. One factor that may contribute to the moderate reduction in clearance of ADMA and SDMA relative to inulin is protein binding. However, the reported binding rates, 8% for ADMA [23] and 9% for SDMA [23], are relatively low and would have only a minor effect on glomerular filtration. Thus, even for these substrates, there appears to be a certain degree of reabsorption from the urine.

### 4.2. Key Candidates for Differential Transport of L-Arginine Derivatives

In the context of reabsorption, particular attention should be given to the transporter b^0,+^AT-rBAT, which is the only known luminal transporter capable of mediating the uptake of L-arginine, L-homoarginine, and ADMA, as demonstrated by Banjarnahor et al. [27] (although data for SDMA transport via b^0,+^AT-rBAT are currently lacking). Notably, for ADMA, no saturation was observed, indicating a substantially lower affinity as compared to L-arginine and L-homoarginine. This difference in affinity may contribute to the observed differences in renal clearance among the three compounds and could help resolve ongoing discussions regarding the mechanism(s) explaining their differential renal handling.

However, reabsorption is not the only factor influencing plasma clearance. A study by Ronden et al. provided further insight, showing that ADMA is not solely dependent on glomerular filtration for its renal elimination but is also absorbed and metabolized from the basolateral (blood-facing) side [25]. In their analysis of CKD cohorts, the renal plasma clearance of SDMA declined by approximately 56% between individuals with eGFR > 90 and those with eGFR 30–59, whereas the decrease for ADMA was only around 26% [25]. This disparity implies either the involvement of basolateral transporters capable of discriminating between ADMA and SDMA or a relatively greater compensatory reserve of metabolic pathways for ADMA as compared to SDMA. Identifying basolateral transporter(s) contributing to a differential uptake remains a challenging objective for future research. The data available, so far, do not present any clear results. Notwithstanding yet unidentified transporters, a contribution of y^+^LAT1-4F2hc and even OCT2 or OATP4C1 cannot be ruled out.

Moreover, we identified only limited (if any) experimental data regarding the regulation of these transport proteins in the renal tubular system. However, regulation of the transport proteins is a further factor to be considered in future research. A detailed discussion of the regulation of the transport proteins in the tubular system is beyond the scope of this review as regulation/modulation of function is highly complex and data are still limited.

The homeostasis of L-arginine, L-homoarginine, ADMA, and SDMA plasma concentrations is linked, among other factors, to kidney function, albeit to a varying degree, reflecting for each substrate the net effect of the relative contribution of reabsorption and secretion mediated by tubular transport proteins as well as synthesis and/or metabolism in tubular cells. Put into perspective, tubular transport is likely to be an important, but not the sole, factor explaining the observed differential renal handling of L-arginine and its derivatives (see exemplary metabolic pathways in Figure 2). A key limiting factor for advancing mechanistic insight is the lack of adequate proximal tubule cell lines that mirror in vivo gene expression. Khundmiri et al. analyzed fourteen different proximal tubule cell lines from six species and found that the commonly used human cell line HK-2 showed only 26% transcriptomic homology to native proximal tubule cells [154]. This significant discrepancy underscores that functional conclusions drawn from commonly used immortalized proximal tubule models must be interpreted with caution, and highlights the importance of validating transporter function in physiologically relevant systems, ideally using primary cells or in vivo models. To facilitate further research, the authors have made these data publicly available in a searchable database (https://esbl.nhlbi.nih.gov/JBrowse/KCT/ (accessed on 6 June 2025)) [154].

Taken together, transport proteins such as b^0,+^AT-rBAT and y^+^LAT1-4F2hc may constitute interesting candidates for pharmacological interventions to modulate the homeostasis and effects of L-arginine-related compounds. Moreover, transporters with clinical evidence for a net impact on the homeostasis of L-arginine derivatives should also be investigated as sites mediating possible adverse drug effects.

## Figures and Tables

**Table 2:** Kinetic parameters for human transport proteins involved in L-arginine and L-arginine derivative transport and associations of diseases with transport function.

Transport Protein	Direction	Counter ion(s)	L-Arginine	L-Homoarginine	ADMA	SDMA
y^+^LAT1-4F2hc	Efflux	Na^+^, neutral amino acids [32,109,110]	K_m_: 182 ± 35 µM [111] V_max_: 3.822 ± 0.24 nmol × mg protein^−1^ × min^−1^ [111] Elevated urine and lowered plasma concentrations in LPI patients [69]	No in vitro data Elevated urine concentrations in LPI patients [69]	No in vitro data Case report of elevated intracellular ADMA concentrations [112]	No in vitro data No clinical data
b^0,+^AT-rBAT	Uptake	Neutral amino acids [82,110]	K_m_: 179.0 µM [113] K_m_: 512.6 ± 109.3 µM [27] V_max_: 1.9 ± 0.1 nmol × mg protein^−1^ × min^−1^ [27] Elevated urine concentrations in cystinuria patients [18]	K_m_: 197.0 ± 31 µM [27] V_max_: 0.7 ± 0.02 nmol × mg protein^−1^ × min^−1^ [27] Elevated urine concentrations in cystinuria patients [18]	K_m_: not detected V_max_: >5 ± 0.5 nmol × mg protein^−1^ × min^−1^ [27] No clinical data	No in vitro data No clinical data
OATP4C1	Uptake and efflux	/	K_m_: 48.1 ± 5.7 µM [29] V_max_: 500.0 ± 19.9 pmol × mg protein^−1^ × min^−1^ [29] No clinical data	K_m_: 49.9 ± 9.6 µM V_max_: 355.7 ± 23.0 pmol × mg protein^−1^ × min^−1^ [29] No clinical data	K_m_: 232.1 ± 78.9 µM [29] V_max_: 351.6 ± 55.0 pmol × mg protein^−1^ × min^−1^ [29] No clinical data but increased *SLCO4C1* mRNA expression was associated with elevated ADMA elimination in rats [114]	K_m_: 70 µM [115] No clinical data
OCT2	Uptake	/	K_m_: >10,000 µM [28] V_max_: >50.0 nmol × mg protein^−1^ × min^−1^ [28] No clinical data	No in vitro data No clinical data	K_m_: 967 ± 143 µM [28] V_max_: 6.3 ± 0.3 nmol × mg protein^−1^ × min^−1^ [28] No clinical data	K_m_: no saturation [115] No clinical data
MATE1	Efflux	H^+^	Substrate [28] No clinical data	No in vitro data No clinical data	Transported [28] No clinical data	K_m_: 1973 µM [115] No clinical data

## Abbreviations

The following abbreviations are used in this manuscript:

ADMA	Asymmetric dimethylarginine
AGAT	Arginine: glycine amidinotransferase
AGXT2	Alanine-glyoxylate aminotransferase 2
b^0,+^AT	b(^0,+^)-type amino acid transporter 1
CAT1	Cationic amino acid transporter 1
CAT2	Cationic amino acid transporter 2
CKD	Chronic kidney disease
CVD	Cardiovascular disease
DDAH1	Dimethylaminohydrolase 1
eGFR	Estimated glomerular filtration rate
HPA	Human Protein Atlas
KTA	Kidney Tissue Atlas
MATE1	Multidrug and toxin extrusion protein 1
NO	Nitric oxide
NOS	Nitric oxide synthase
OATP4C1	Organic anion transporting polypeptide 4C1
OCT2	Organic cation transporter 2
scRNA	Single-cell RNA
SDMA	Symmetric dimethylarginine
SNP	Single nucleotide polymorphisms
y^+^LAT1	y^+^L amino acid transporter 1
y^+^LAT2	y^+^L amino acid transporter 2
